# On the Use of Oxide of Silver

**Published:** 1852-06

**Authors:** J. V. Withers

**Affiliations:** Brandenburg, Ky.


ART. IV.—*On the Use of Oxide of Silver.* By J. V. WITHERS, M. D., of Brandenburg, Ky.

I look upon this preparation of silver as being possessed of far greater medicinal properties than medical authorities generally ascribe to it.

The United States' Dispensatory places it where it but rarely falls under the notice of the reader. Dunglison on *New Remedies* gives it a more prominent position; but you may travel for days, and not find a grain of it in the physician's shop. Speak to them on the subject of its medicinal properties, and their ideas are vague and confused, many having not more than listened to or casually glanced over its name and history. Now I do not expect that my pen will give it any superior claims to the profession, or introduce it into general use; but I wish to add my experience and observations respecting its qualities as a curative agent, and to ask of practitioners a more general investigation of its merits.

I have been using it in my practice for about three years, and I must say it has come as near if not nearer producing the effects desired, with fewer failures, than any one remedy I have employed in that time.

What are the indications for its employment? It has been more frequently used in the complaints of females—those cases at or about the change of life, where there is great derangement of the digestive organs, pain on pressure over the epigastrium, a constant burning in the stomach, febrile excitement at night, slight redness of the tongue, eructations, horripilations, &c. In these cases the bowels are subject to too great laxness, alternating with constipation; uterine hemorrhage every two, four, or six weeks. Given in half grain doses, made into pills, three times a day, one hour before eating, the oxide of silver has not failed, in a single instance, of relieving or greatly mitigating the sufferings of the patient in such cases.

They commence improving immediately, when, in many instances, other remedies had to all appearance aggravated their condition. I have contined the remedy until as much as 40, 60, and even 80 grs. have been given. The uterine hemorrhage is invariably moderated, the stomach and bowels improved or restored to their functions; sleep returns, and the patient again becomes cheerful, whereas before she was gloomy and morose, constantly dwelling on her internal uneasy sensations.

The mucous membrane of the stomach is clearly in a state of sub-acute inflammation, in such cases; the disorder of the bowels, there is reason to believe, is primarily of a similar character; or it may be that a secondary functional derangement results from the failure of the stomach to perform its function. The lining membrane of the uterus is doubtless in a like situation. I give the Oxide of silver to remove these morbid conditions, and, as I have already remarked, it has never failed to do so in a triumphant manner. I hold that it must act as an alterative, and especially upon the mucous membranes. In what manner it acts I can no more explain than I can tell how quinine will cut short a quotidian, or how mercury arrests the development of fibrine in the blood. They are ultimate facts which rest upon enlarged experience and observation.

The result of my experience is, that the oxide is superior to the nitrate of silver, and may be prescribed with great advantage in those cases of chronic dysentery, and diarrhea, in which the latter has been recommended by writers.

January, 1852.

